# An insulin resistance associated neural correlate of impulsivity in type 2 diabetes mellitus

**DOI:** 10.1371/journal.pone.0189113

**Published:** 2017-12-11

**Authors:** Kristen L. Eckstrand, Nishit Mummareddy, Hakmook Kang, Ronald Cowan, Minchun Zhou, David Zald, Heidi J. Silver, Kevin D. Niswender, Malcolm J. Avison

**Affiliations:** 1 Department of Radiology, Vanderbilt University Medical Center, Nashville, TN, United States of America; 2 Department of Biostatistics, Vanderbilt University Medical Center, Nashville, TN, United States of America; 3 Department of Psychiatry, Vanderbilt University Medical Center, Nashville, TN, United States of America; 4 Department of Psychology, Vanderbilt University Medical Center, Nashville, TN, United States of America; 5 Department of Medicine, Vanderbilt University Medical Center, Nashville, TN, United States of America; VU University Medical Center, NETHERLANDS

## Abstract

Central insulin resistance (IR) influences striatal dopamine (DA) tone, an important determinant of behavioral self-regulation. We hypothesized that an association exists between the degree of peripheral IR and impulse control, mediated by the impact of IR on brain circuits controlling the speed of executing “go” and/or “stop” responses. We measured brain activation and associated performance on a stop signal task (SST) in obese adults with type 2 diabetes (age, 48.1 ± 6.9 yrs (mean ± SD); BMI, 36.5 ± 4.0 kg/m^2^; HOMA-IR, 7.2 ± 4.1; 12 male, 18 female). Increasing IR, but not BMI, was a predictor of shorter critical stop signal delay (cSSD), a measure of the time window during which a go response can be successfully countermanded (R^2^ = 0.12). This decline was explained by an IR-associated increase in go speed (R^2^ = 0.13) with little impact of IR or BMI on stop speed. Greater striatal fMRI activation contrast in stop error (SE) compared with stop success (SS) trials (CON_SE>SS_) was a significant predictor of faster go speeds (R^2^ = 0.33, p = 0.002), and was itself predicted by greater IR (CON_SE>SS_ vs HOMA-IR: R^2^ = 0.10, p = 0.04). Furthermore, this impact of IR on striatal activation was a significant mediator of the faster go speeds and greater impulsivity observed with greater IR. These findings suggest a neural mechanism by which IR may increase impulsivity and degrade behavioral self-regulation.

## Introduction

Insulin receptors are broadly distributed throughout the brain [1, 2], and pre-clinical and human subject studies support the idea that beyond its role as a satiety signal, insulin signaling in the brain modulates the activity of brain regions and networks subserving a diverse répertoire of cognitive, executive, reward and motor functions involved in, but not limited to feeding behavior [3–9] (and for reviews, see [10, 11]). Obesogenic diets and obesity are strongly associated with insulin resistance (IR) and type 2 diabetes mellitus (T2DM) and there is compelling evidence for development of a phenotype of central insulin resistance [7, 11, 12] that may precede [13, 14], and even drive the development of peripheral IR.

Central insulin signaling plays an important role in maintaining optimal dopamine (DA) tone in ventral and dorsal striatum by tuning rates of synaptic DA release [15] and clearance [16, 17], and impairments in central insulin signaling have a direct impact on brain DA systems [17–22]. This and the central role of DA signaling in attention, cognition and impulsivity [23–29], suggest impairments in central insulin signaling as a possible proximal cause of the reports of diabetes mellitus-associated deficits in behavioral self-regulation [30–33] and further, may explain the mixed findings with respect to obesity-associated deficits [34–47] (and see recent review by Bartholdy et al 2016 [48]).

Thus, we hypothesized that increased psychomotor speed and/or poorer inhibitory control would be associated with increased IR, and further, that this association would be mediated by increased neural activity in brain areas identified as substrates of salience attribution, cue reactivity, motor speed, and/or decreased activity in those implicated in inhibitory control. To test the viability of this hypothesis we sought, in a cohort of obese T2DM volunteers in which there was no correlation between severity of IR and BMI, to identify neural predictors of psychomotor speed, impulsivity, and inhibitory control capacity and to determine to what extent the sensitivity of these predictors to IR mediated inter-individual differences in performance.

## Materials & methods

The Vanderbilt University IRB approved this study. Written consent was obtained from all participants.

### Participants

Participants were right handed male and female adults (BMI 30–50 kg/m^2^) aged 31–60 years, with type 2 diabetes mellitus (T2DM; Hemoglobin A1c [HbA1c] 6–8%), stable body weight for three months, no prior insulin treatment, no contraindications for MRI, and no other significant medical conditions or history (tobacco use in previous 3 months, substance abuse or dependence (DSM-IV), current Axis I disorders (DSM-IV), centrally acting medications other than SSRIs in previous year, weight loss surgery, ongoing use of dietary or weight loss supplements; women only: post-menopausal, pregnant; polycystic ovarian disease). Most participants (45/47) were using metformin alone for glycemic control.

### Study protocol

Prior to the study visit, participants practiced the fMRI stop signal task (SST) in a mock MRI scanner until their performance measures were stable. For the imaging study visit, participants arrived at the Vanderbilt Clinical Research Center (CRC) at 1:30 pm for an overnight stay, having abstained from alcohol, caffeine, and physical exercise for 48 hours, and consuming breakfast and a light lunch before 12 pm, and consuming only water until conclusion of the day’s imaging. Following full clinical and metabolic workup, fasting participants were brought to the MRI suite, re-screened for MRI contraindications, re-practiced the SST, and underwent structural and functional neuroimaging, beginning at ~4:00 pm. Upon completion of all studies, participants returned to the CRC, consumed an evening meal, then fasted until 10:00 am, when blood samples were collected for measurement of fasting plasma insulin and glucose. Blood samples were processed immediately and plasma was frozen and stored at -80°C for insulin and glucose analysis.

### fMRI

#### Stop signal task

Participants completed three fMRI runs of the SST design used by Li et al. [49], each lasting ~10 minutes and consisting of 75 go trials and 25 stop trials (3:1 go:stop) presented in pseudo-random order (Figure A in S1 File). Go trials began with a yellow fixation dot fore-period (pseudo-randomized 1–5 seconds) followed by a green circle that served as the go cue for participants to respond with a right index finger button press. The green circle disappeared following a button press or after 1s had passed, whichever came first. Go trials where the button press was premature, later than 1s, or absent, were considered incorrect. Stop trials were the same as go trials except that a red X, serving as the stop cue, appeared after a variable stop signal delay (SSD). Upon the appearance of the red X, participants tried to withhold their response. There was a fixed 2s interval between trials. E-Prime Software v2.0 (Psychology Software Tools) controlled stimulus presentation and response monitoring.

#### SST analysis

Percentage of successful go and stop trials, and median go response times (mGRT) for successful go trials were calculated for each participant. Using the approach of Li and colleagues [49], custom Matlab (Mathworks) code was used to calculate the critical stop signal delay (cSSD, the time interval between go and subsequent stop signal for which 50% of go responses can be successfully countermanded) and the stop signal response time (SSRT = mGRT-cSSD, time required to successfully complete the countermanding stop process). Participants’ SST results were included in the analyses only if they met performance thresholds previously shown to robustly characterize behavioral components of the SST [50, 51]: successful inhibition on 25–75% of stop trials and >60% response rate on go trials. We also confirmed that consistent with the race model assumptions, participants’ stop error response times were shorter than their mGRT.

#### Image acquisition

Images were acquired on a 3T Phillips Achieva MRI Scanner using an 8-channel head coil. 3D T1-weighted TFE gradient echo anatomical images (isotropic 1mm^3^) were collected with 5° flip angle, TI/TR/TE = 959.74/8.3/3.9 ms, in 170 volumes. T2*-Weighted Gradient FFE echoplanar BOLD fMRI images (TR/TE = 2000/35 ms, 79° flip angle, SENSE factor = 1.8, 3x3x4.5mm^3^ voxel size interpolated to 1.8x1.8x4.95mm) were acquired parallel to the AC-PC line.

#### fMRI analysis

Analyses used SPM8. Data were motion corrected, slice-time corrected, and high-pass temporally filtered with a cutoff of 128 sec. Participants exceeding strict motion parameters (2° rotation, 2mm translation) were removed from future analysis. The mean functional image from the slice time correction was then coregistered with the high-resolution 3D anatomic image, normalized to MNI space, and spatially smoothed (Gaussian kernel 6.0 mm FWHM) [52].

Four types of trial outcomes were modeled: go success (GS), go error (GE), stop success (SS), and stop error (SE). Trial onset and reaction times, together with motion covariates, were entered into a statistical design matrix for each participant using a general linear model (GLM). Event onsets associated with each trial type were convolved with a canonical hemodynamic response function and used to construct first-level contrasts.

To screen for candidate neural mediators of SST performance, we generated contrasts between successful and unsuccessful stop trials (SS>SE, SE>SS) and between successful stop and go trials (SS>GS) to identify volumes of interest (VOIs) in which mean contrast could be regressed against SST performance. As these initial screens for candidate mediators were exploratory, we used a relatively relaxed threshold (p_unc_ < 0.001, k_E_ = 10), chosen to balance sample size, anatomic specificity, and clinical significance [53]. Candidate VOIs identified by these screens were used to test for an association of activation strength with SST performance parameters across participants. We also performed whole brain voxel-level regression of contrast strength against SST performance parameters to independently and directly screen for brain areas in which contrast strength was a predictor of SST performance, and/or was itself predicted by BMI and/or HOMA-IR (p_unc_<0.005, k_E_ = 10).

Marsbar (http://marsbar.sourceforge.net/) was used to calculate the individual participant weighted parameter estimates of the activity within the VOIs [54].

### Central insulin resistance

While central IR in the absence of significant peripheral resistance has been observed in the setting of Alzheimer’s disease, the preponderance of available data suggest a reasonable correlation of central with peripheral IR in T2DM [7, 12]. We therefore used the homeostatic model assessment of insulin resistance (HOMA-IR) score [55], based on fasting plasma insulin and glucose concentrations, as a surrogate reporter of central IR. Metformin was the sole form of glycemic control for almost all participants (45/47), and metformin dose was therefore included as a covariate in all analyses.

### Statistical analyses

All statistical analyses were performed using SPSS v22.0.0.

#### Behavioral data

Sensitivity of SSRT, cSSD and/or mGRT to obesity (BMI) and HOMA-IR, was assessed using multivariate regression with BMI and HOMA-IR as independent predictors, with age, sex, and metformin dose included as covariates. Absent evidence of any BMI x HOMA-IR or HOMA-IR x metformin interactions, none were included in the final models.

#### fMRI data

Analysis of fMRI data comprised tests for associations of SST performance with strength of neural activation on the one hand, and associations of neural activation strength with degree of obesity and/or insulin resistance on the other. Neural predictors of SST performance were identified by multivariate regression of activation contrasts against individual SST performance parameters, with age, sex, and metformin dose included as covariates. Similarly, metabolic predictors of activation were identified by multivariate regression of activation contrasts using BMI and HOMA-IR as independent predictors. Age, sex, and metformin dose were included as covariates in initial analyses, and were retained in the final model if they were significant contributors to overall variance. As for behavioral analyses, absent evidence of any BMI x HOMA-IR or HOMA-IR x metformin interactions, none were included in the final models.

#### Mediation analysis

A bootstrapped mediation model (PROCESS v 2.13, Hayes; implemented in SPSS v 22.0.0) was used to determine the degree to which the impact of BMI or HOMA-IR on activation of specific brain areas mediated any association of stop signal performance with BMI or HOMA-IR. Within this model we examined both the direct and indirect mediation effects. Absence of a zero crossing in the 95% confidence interval for the mediating path coefficient, together with κ^2^ ≥ 0.1 was taken as evidence for mediation.

## Results

### Demographics and clinical information

All forty-seven participants (18 male / 29 female; age, 46.7 ± 6.9 yrs (mean ± SD), range 31–60; BMI, 37.0 ± 4.5 kg/m^2^, range 28.7–49.8; HOMA-IR, 8.2 ± 4.9, range 2.5–25.6; HbA1c, 7.1 ± 1.4, range 5.8–9.2) met the SST performance criteria and were included in behavioral analyses. Of these, a subset of thirty (age, 48.1 ± 6.9 yrs, range 36–60; BMI, 36.5 ± 4.0 kg/m^2^, range 28.7–45.1; HOMA-IR, 7.2 ± 4.1, range 2.5–17.9; HbA1c, 7.3 ± 0.8, range 5.9–9.1; male/female, 12/18) met the more stringent head motion criteria for inclusion in the fMRI analyses. This fMRI sub-group did not differ significantly from the parent group in clinical measures, including age, BMI, HOMA-IR or HbA1c, though the fMRI subgroup trended marginally older in age, and lower in HOMA-IR. Demographic and clinical information for the parent and fMRI sub-groups are summarized in Table 1.

**Table 1:** Demographic and clinical data.

	Parent (Behavioral) Group		fMRI Sub-group	p_mean_ ^a^	p_var_ ^a^
N	47	30		-	
Sex	29F, 18M	18F, 12M		-	
Race ^b^	20B, 2H, 25W	11B, 1H, 18W		-	
Education (yrs)	14.8 ± 2.0	15.07 ± 2.0		0.926	
Age (yrs) ^c^	46.7 ± 6.9 [40.0 48.0 51.0]	48.1 ± 6.9 [41.8 49.0 52.5]		0.06	0.78
BMI (kg/m2) ^c^	37.0 ± 4.5 [33.9 36.7 40.5]	36.5 ± 4.0 [33.6 36.8 40.3]		0.21	0.11
HOMA-IR ^c^	8.2 ± 4.9 [5.0 6.7 10.1]	7.2 ± 4.1 [4.4 6.3 8.5]		0.07	0.27
Fasting Glucose (mg/dl) ^c^	133 ± 41 [103 128 153]	129 ± 41 [102, 120, 144]		0.42	0.95
Fasting Insulin (μu/ml) ^c^	24.2 ± 11.4 [16.1 22.5 28.6]	22.3 ± 9.9 [14.8 21.3 23.5]		0.11	0.3
HbA1c ^c^	7.1 ± 1.4 [6.6 7.2 7.8]	7.3 ± 0.8 [6.6 7.3 7.8]		0.23	0.13
				

**Parent (Behavioral) Group:** volunteers whose SST performance met criteria for inclusion in behavioral analyses (see Methods).

**fMRI subgroup:** volunteers meeting SST performance criteria whose fMRI head motion met criteria for inclusion in fMRI analyses. All data mean ± SD.

^**a**^ probability of difference in mean, variance, between parent cohort and fMRI subgroup

^**b**^ B—black, H—Hispanic, W–white

^**c**^ continuous variables reported as mean +/- sd, [25^th^ 50^th^ 75^th^] percentiles

### Correlations amongst metabolic factors

Participants’ BMI and HOMA-IR were not correlated whether controlling for age, metformin or sex (R^2^ = 0.04, p = 0.21 parent behavioral cohort; R^2^ = 0.007, p = 0.68 fMRI subgroup of parent cohort) or not (R^2^ = 0.04, p = 0.19 behavioral; R^2^ = 0.003, p = 0.77 fMRI), allowing us to examine the association of behavioral and neural responses with IR and obesity independently. The low correlation between HOMA-IR and BMI in both parent cohort and fMRI subgroup occurred despite broad and relatively even sampling of HOMA-IR and BMI, as judged by their respective inter-quartile ranges (Table 1). Nonetheless, the absence of lean volunteers in this study, which focused on volunteers with T2DM, may have reduced our power to detect associations of BMI per se with behavior and/or neural response.

Consistent with the absence of any correlation between BMI and HOMA-IR, we also found no correlation of fasting plasma glucose or insulin with BMI.

### Metabolic predictors of SST performance

SST performance was similar to that previously observed in comparable healthy control participants [45, 49, 56]: cSSD (310 ± 119 ms; mean ± SD), mGRT (605 ± 107 ms), SSRT (295 ± 32 ms), was not significantly different in the fMRI subgroup, and did not differ significantly between male and female participants in either case (Table A in S1 File). Consistent with the race model premise [57, 58], participants’ response times for SE trials were shorter than for GS trials.

**Fig 1:**
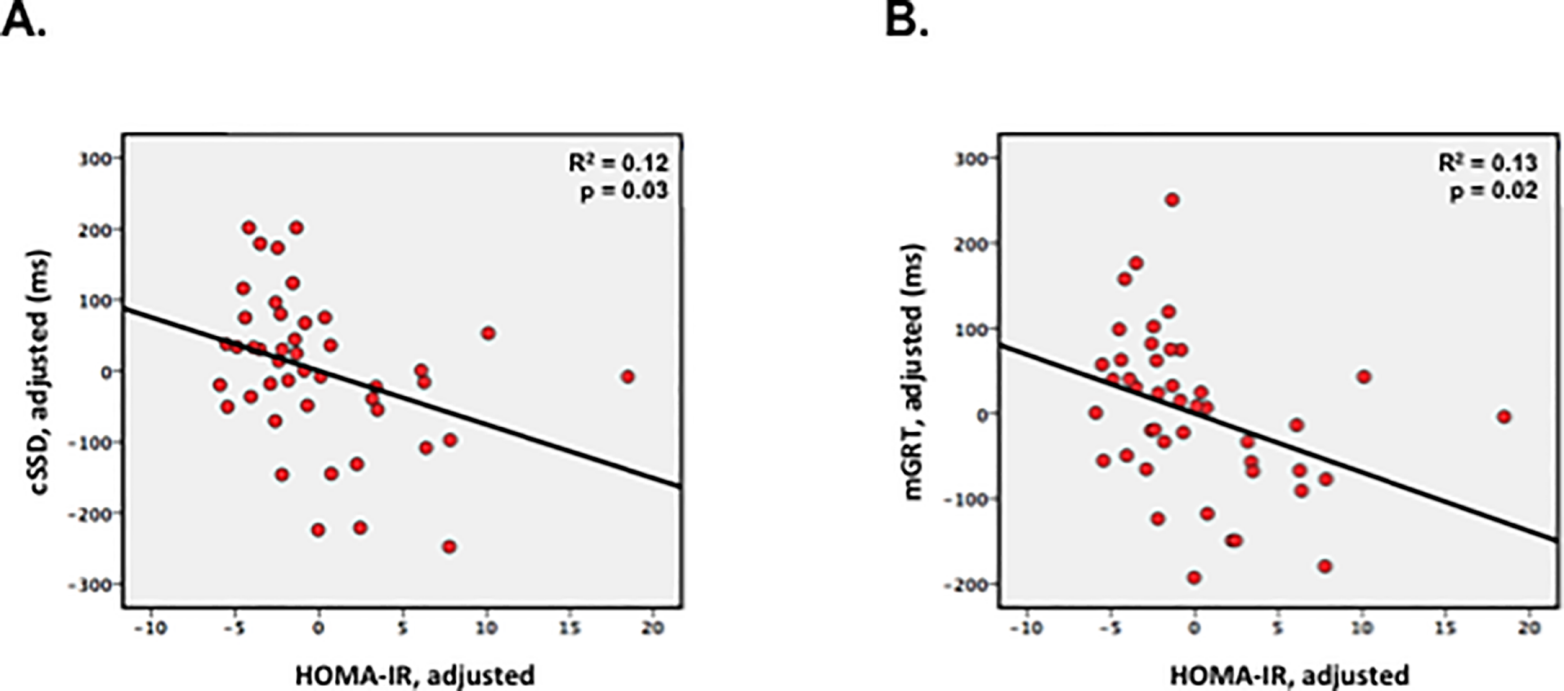
HOMA-IR is the dominant predictor of overall Stop Signal Task performance in obese T2DM participants. **A)** Increasing insulin resistance (HOMA-IR) predicts poorer overall performance (shorter critical stop signal delay, cSSD). **B)** This degradation in SST performance is a consequence of faster go speeds (shorter mGRT) with increasing insulin resistance. Models include age and metformin dose as covariates.

BMI was not a predictor of any component of SST performance, but HOMA-IR was: cSSD (r = -0.35, p = 0.03) and mGRT (r = -0.36, p = 0.02) decreased with increasing HOMA-IR (Fig 1; covariates: BMI, age, metformin). Interestingly, metformin dose was also a significant independent predictor: cSSD (r = -0.46, p = 0.003) and mGRT (r = -0.47, p = 0.002) decreased with increasing metformin dose. HOMA-IR was not a predictor of SSRT however, suggesting that IR reduces cSSD by accelerating go speed.

Because of the co-linearity of HOMA-IR with fasting plasma glucose (FPG) and insulin, we tested each of these as independent individual predictors of neural activation. Consistent with the association of shorter cSSD and mGRT with increasing HOMA-IR, higher FPG was also a significant predictor of shorter cSSD (r = -0.40, p = 0.01; covariates: BMI, age, metformin) and mGRT (r = -0.34, p = 0.03; covariates: BMI, age, metformin), although fasting plasma insulin was not.

### Neural predictors of SST performance

**Fig 2:**
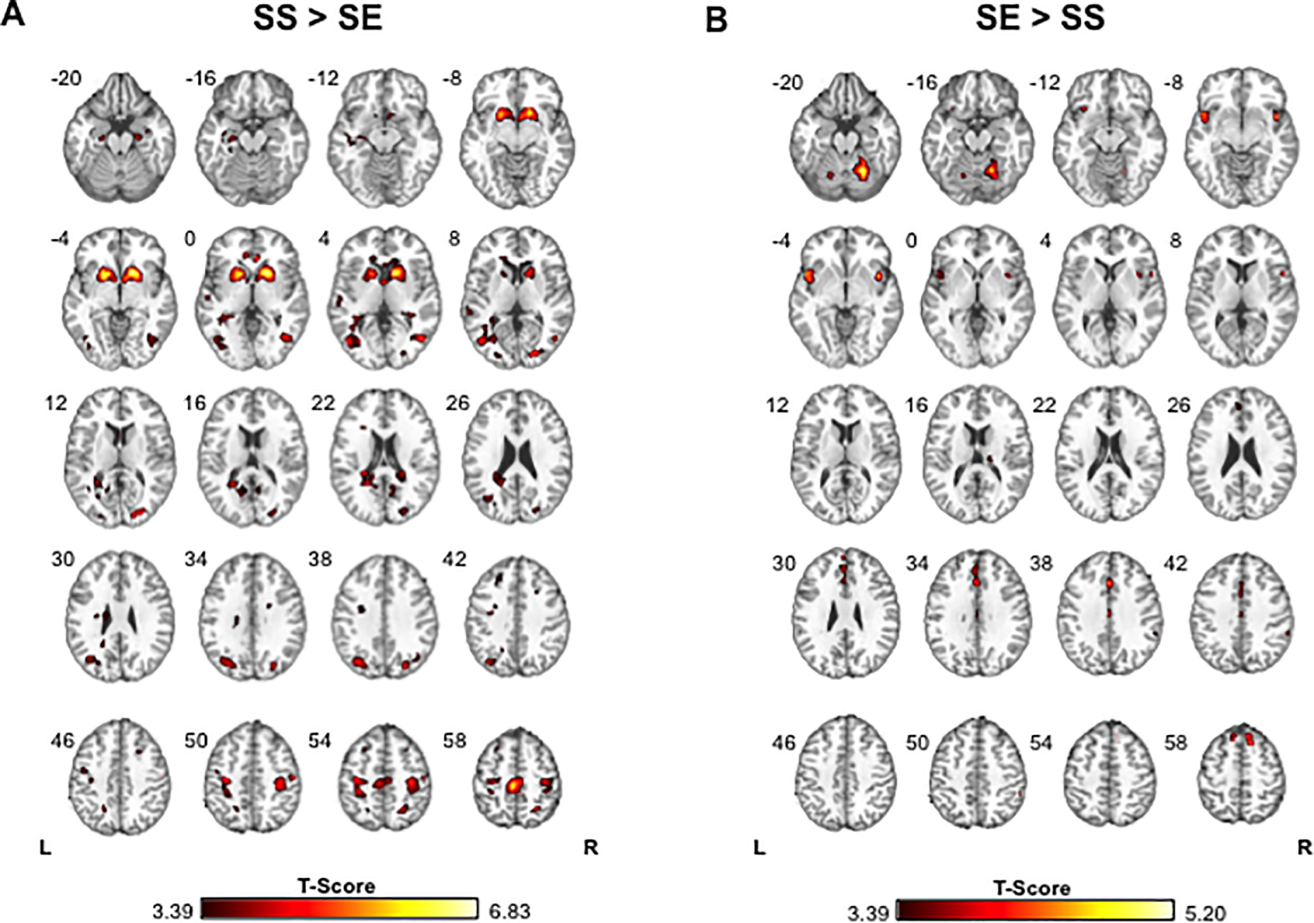
Group contrast T-maps comparing activity between stop success (SS) and stop error (SE) trials, thresholded at p_unc_<0.001, k_E_>10 voxels. **A)** SS>SE reveals greater activation in SS compared with SE trials in bilateral dorsal striatum, precentral gyrus, supplementary motor and visual areas, as well as the right cingulate gyrus, temporal gyri, and right superior parietal lobule. **B)** SE>SS reveals greater activation for stop error trials in bilateral cingulate gyrus, medial frontal gyrus/preSMA, insula, and cerebellum.

Consistent with previous studies of response inhibition [49, 59–62], we observed greater bilateral activation of elements of inhibitory networks [49, 61–64] for SS>SE trials, including striatum, precentral (M1) and supplementary (SMA) motor cortex (Fig 2A, Table 2). SE>SS revealed greater bilateral activation of insula, medial frontal gyri (MeFG)/preSMA, cingulate gyrus and cerebellum, areas implicated in attention, salience attribution, performance/error monitoring and motor speed (Fig 2B, Table 2).

**Table 2:** Brain areas showing significant differences in activation between stop success (SS) and stop error (SE) trials.

Contrast	Brain Region	Hemi.	Voxels	Max. T-statistic	x	y	z
	Anterior Cingulate Gyrus	R	13	5.5957	6	32	-2
	Caudate/Putamen	L	147	6.4413	-18	11	-5
		R	196	6.8297	18	14	-2
	Hippocampus	L	13	4.6395	-33	-43	1
	Middle Occipital Gyrus	L	57	5.6398	-42	-70	7
		R	44	5.614	21	-85	7
SS > SE	Middle Temporal Gyrus	R	37	5.3596	45	-67	4
	Parahippocampal Gyrus	L	10	4.103	-24	-19	-17
	Postcentral Gyrus	R	11	4.5868	54	-16	49
	Precentral Gyrus	L	54	5.9114	-33	-19	49
		R	85	4.9799	33	-28	49
	Precuneus	L	54	4.9169	-30	-73	34
		R	17	5.4281	27	-76	34
	SMA	L/R	264	5.8732	-3	-25	58
	Superior Parietal Lobule	R	16	5.3096	24	-55	55
	Cerebellum	L	14	3.7239	-36	-58	-26
		R	75	5.1984	21	-58	-20
SE > SS	Cingulate Gyrus	L/R	17	4.5074	0	23	34
	Insula	L	37	4.5641	-45	5	-5
		R	18	4.94	42	8	-5
	preSMA / Medial Frontal Gyrus	R	37	4.8283	12	20	61
		L	11	4.3431	-9	26	58

All contrasts generated using p_unc_<0.001 and k_E_>10.

**Fig 3:**
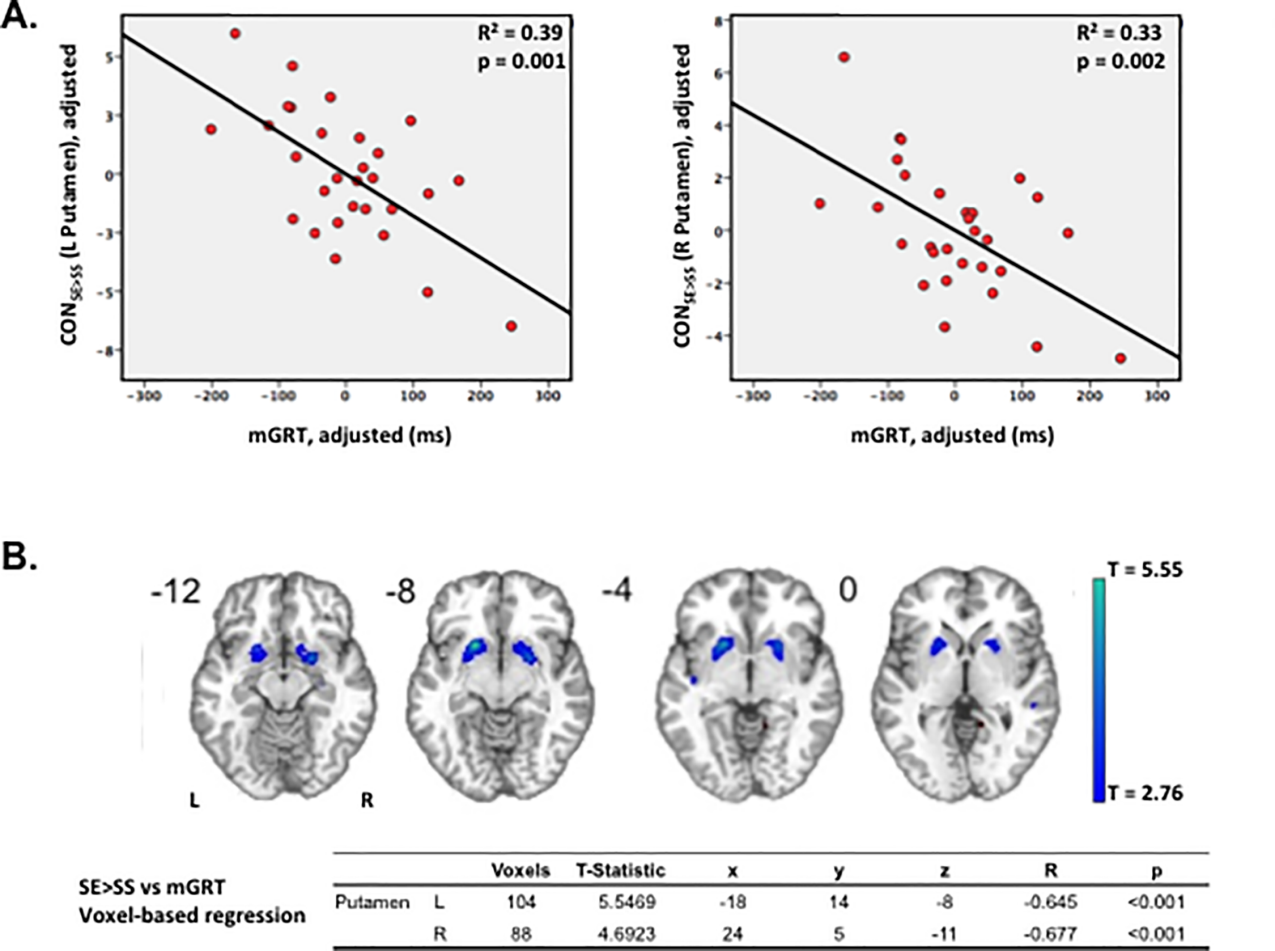
Activation in bilateral striatum predicts faster go speeds. **A)** Significant association of faster go speeds (shorter mGRT) with greater activation contrast for failed vs. successful stop trials (CON_SE>SS_) in bilateral striatum, consistent with greater “go”-associated putamen activation in fast responders. (Striatal ROIs extracted from group SS>SE contrast map, Fig 2A). **B)** Whole brain voxel-wise regression of CON_SE>SS_ against mGRT confirms a strong negative correlation in putamen bilaterally. (Map thresholded at an uncorrected p<0.005).

Surprisingly however, in none of the putative inhibitory areas identified by SS>SE did contrast strength (CON_SS>SE_) predict SSRT. Rather, CON_SS>SE_ in putamen was a predictor of longer mGRT that, given the lack of association with SSRT, can be reframed as an association of CON_SE>SS_ with shorter mGRT, consistent with greater “go”-associated putamen activation in fast responders (Fig 3A). Whole brain voxel-wise regression of participants’ CON_SE>SS_ against mGRT confirmed the ROI-based association (Fig 3B).

SS>SE and SE>SS contrasts are well controlled with respect to trial type, attentional demand, sensory processing, behavioral response set, and shift of response set. However differences in level of activation of inhibitory circuits between SS and SE trials may be small, diminishing the sensitivity of this contrast to earlier components of inhibition [60]. Conversely, SS>GS has greater sensitivity to, but less specificity for inhibitory processing. Consistent with this, voxel-level regression across the whole brain identified several areas where CON_SS>GS_ was a significant predictor of mGRT or SSRT (Figure B, Table B in S1 File). In a network comprising largely right hemisphere and bilateral structures (right MFG, PCG, angular gyrus, bilateral cuneus, thalamus; left SMA), increasing activation predicted shorter mGRTs, while the opposite was the case for a predominantly left hemisphere network (left putamen, MTG, IFG, insula; right amygdala), in which increasing activation was associated with longer mGRTs. Thus increased activation of the right hemisphere network appears to promote faster responding to “go” cues, while increasing activation of the left hemisphere network is associated with slower responding. Interestingly, we observed a congruent pattern of brain-behavior correlates for SSRTs: increased activation in a left frontal cortical network (IFG, MFG, SFG) predicted shorter SSRTs, while greater right hemisphere activation in insula and supramarginal gyrus was associated with longer SSRTs (Figure B, Table B in S1 File).

### Impact of metabolic factors on neural correlates of SST performance

**Fig 4:**
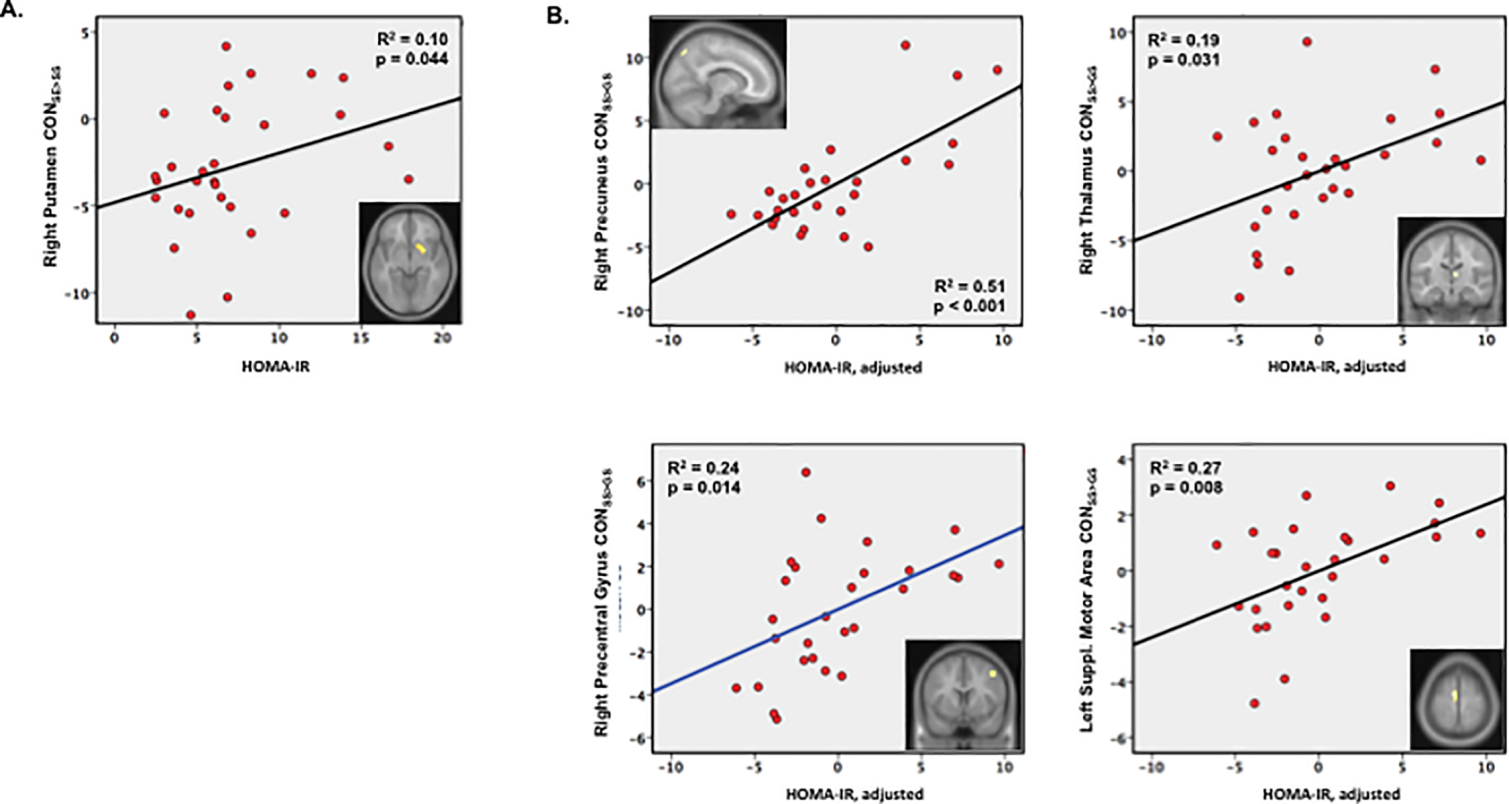
Insulin resistance predicts brain activation in brain regions whose activities are themselves predictors of mGRT. **A)** Increasing HOMA-IR predicted decreasing activation for successful vs failed stop trials (CON_SS>SE_) in right putamen, where lower contrast predicted faster go speeds (shorter mGRT). **B)** Increasing HOMA-IR predicted increasing activation for go compared with stop success trials (CON_SS>GS_) in right precuneus, right thalamus, left supplementary motor area, and right precentral gyrus, brain areas where greater contrast predicted faster go speeds.

Consistent with the faster go speeds observed in participants with higher fasting plasma glucose and greater insulin resistance, FPG and/or HOMA-IR were significant predictors of activation in several areas identified as neural correlates of mGRT: increasing FPG (r = -0.35, p = 0.03) and HOMA-IR (r = -0.31, p = 0.04; Fig 4A) both predicted increasing activation of right putamen in failed compared with successful stop trials (CON_SE>SS_), and increasing CON_SE>SS_ was itself a predictor of faster go speeds. Similarly, increasing FPG and HOMA-IR (Fig 4B) predicted greater CON_SS>GS_ activation for four areas in which such an increase was also a predictor of shorter mGRT: right precuneus (FPG r = 0.45, p = 0.03; HOMA-IR r = 0.71, p < 0.001), right thalamus (FPG r = 0.42, p = 0.04; HOMA-IR r = 0.43, p = 0.031), right precentral gyrus (FPG r = 0.47, p = 0.03; HOMA-IR r = 0.49, p = 0.014), and left SMA (FPG r = 0.48, p = 0.014; HOMA-IR r = 0.52, p = 0.008).

**Fig 5:**
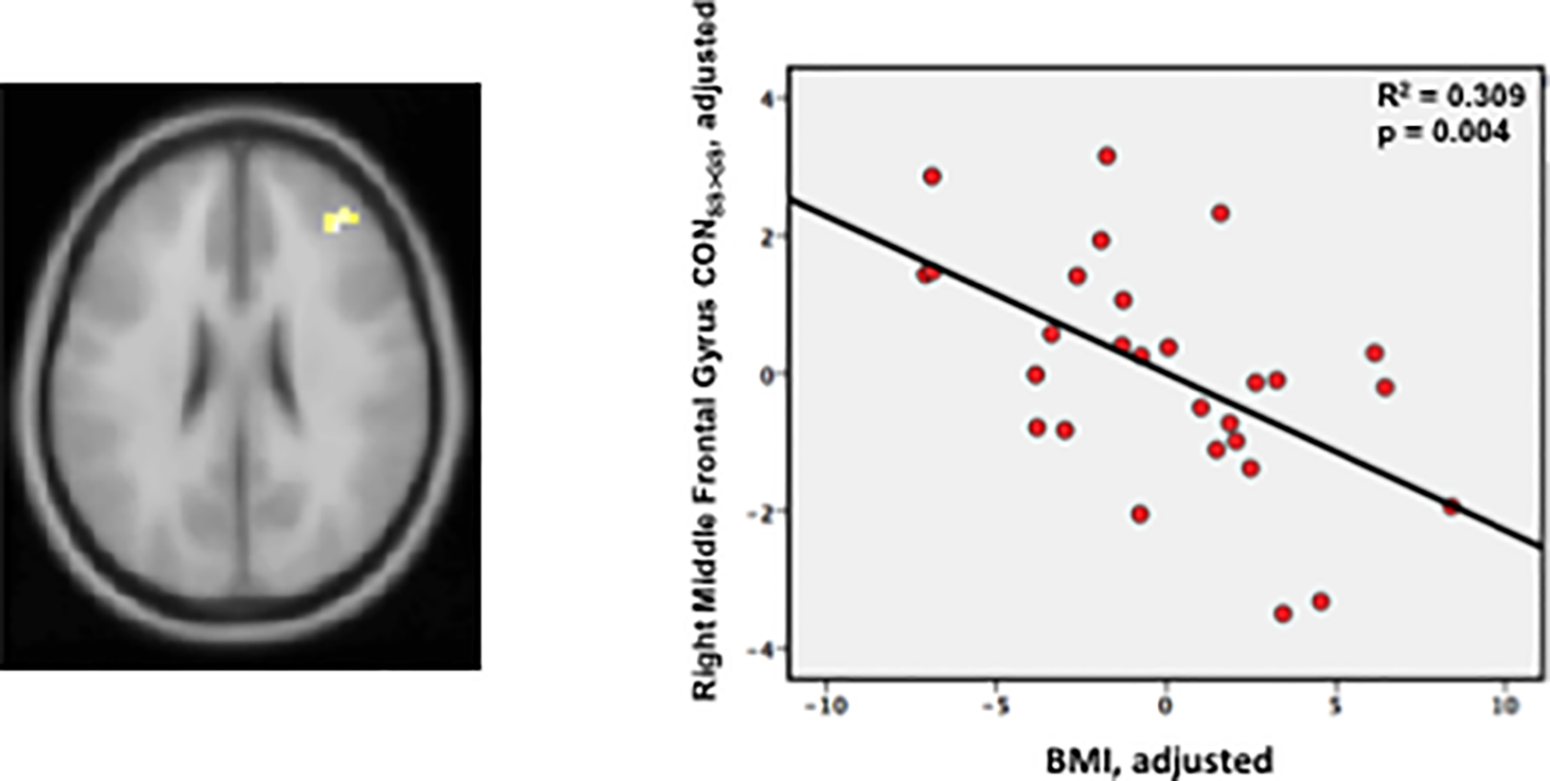
BMI predicts activation in right middle frontal gyrus. Decreasing CON_SS>GS_ activation with increasing BMI in right middle frontal gyrus, an area whose activation strength is a significant predictor of go speed (i.e. increasing activation predicted shorter mGRT, see Table B, Figure B panel A, in S1 File).

Though we found no association of SSRT or mGRT with BMI, we nonetheless tested for evidence that BMI might predict strength of activation in areas identified as neural predictors of SST performance. Consistent with BMI’s failure to predict any aspect of SST performance, it also failed to predict activation contrast for any neural predictors of SSRT. Higher BMI was, however, associated with smaller SS>GS activation in right MFG (CON_SS>GS_ vs BMI: r = -0.56, p = 0.004; covariates: BMI, age, metformin; Fig 5), itself a predictor of longer mGRT.

### Impact of HOMA-IR on neural activation mediates heightened impulsivity with increasing insulin resistance

**Fig 6:**
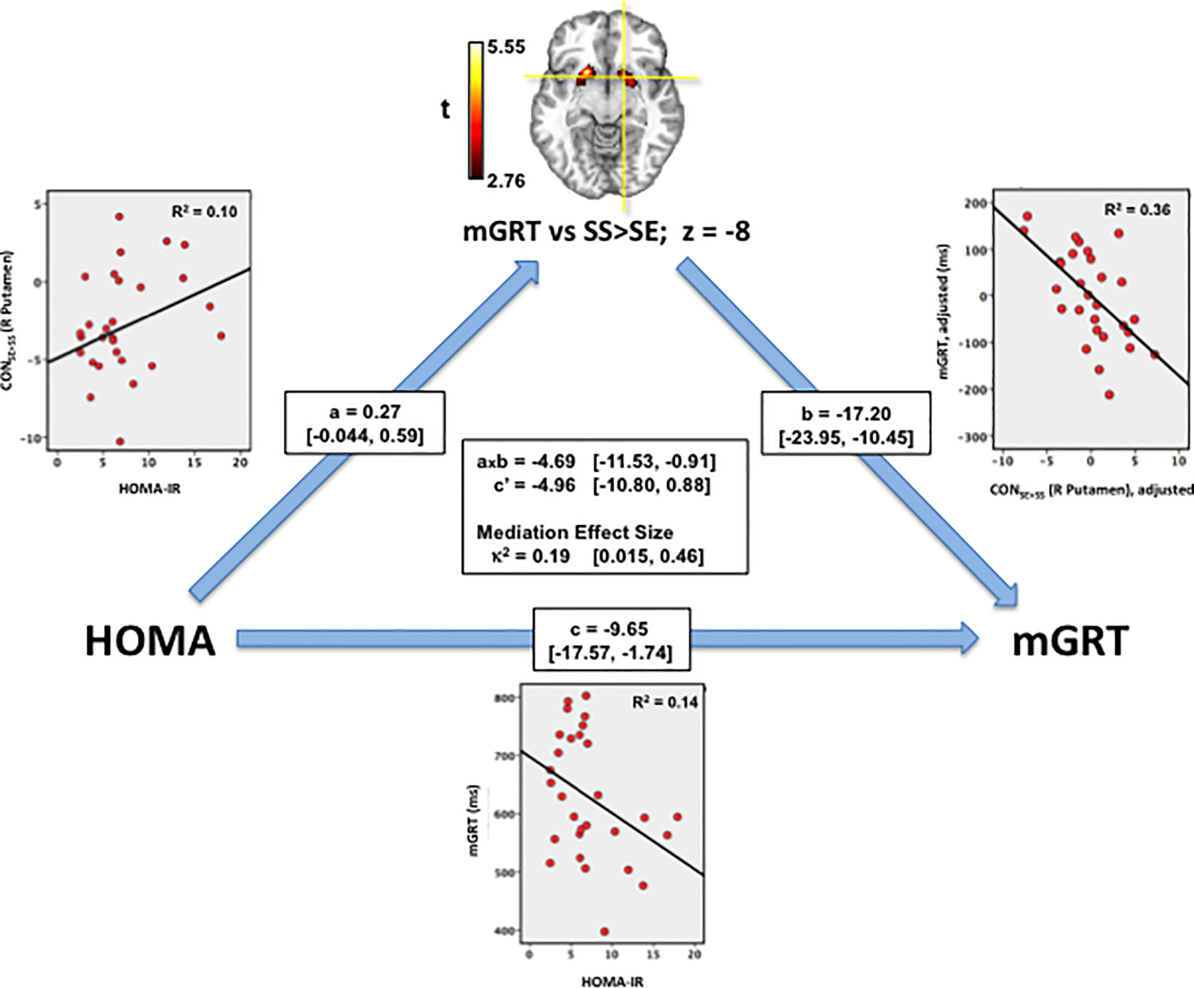
Impact of insulin resistance on striatal activation mediates impulsivity in T2DM. Through an association of increasing activation (CON_SE>SS_) with increasing insulin resistance (path a: HOMA-IR -> CON_SE>SS_), putamen activation, a predictor of faster go speeds (shorter mGRT; path b: CON_SE>SS_ -> mGRT) mediates the faster go speeds (shorter mGRT) observed with greater insulin resistance (path c: HOMA-IR -> mGRT) observed in obese T2DM participants with a medium to large effect size (κ^2^ 0.19, 95% CI = 0.015–0.46. Bootstrapped path coefficients a, b, c, c’ and [95% confidence intervals] determined using PROCESS in SPSS 22.

Finally, we determined whether any of the brain regions identified as neural predictors of mGRT mediated the association of faster go speeds with increasing insulin resistance. Path analysis [65] confirmed the association of right putamen activation with HOMA-IR as a significant mediator of the faster go speed (shorter mGRT) observed with greater insulin resistance, with a medium to large mediating effect size (κ^2^ = 0.19, range [0.02–0.46]; Fig 6).

It also provided trend level support for a similar mediating role for the impact of insulin resistance on a network of brain areas including right precentral gyrus, precuneus and thalamus, and left SMA (Figure C in S1 File).

A similar analysis for FPG found that with the exception of left SMA (κ^2^ = 0.13, range [0.02–0.32]), the association of shorter mGRT with increasing FPG was not significantly mediated by an impact of FPG on regional brain activation.

These results suggest that insulin resistance may heighten impulsive responding through its impact on a network of cortical and subcortical brain areas subserving attentional salience processing, and motor speed.

## Discussion

Our principal finding is that in obesity-associated T2DM inter-individual differences in overall impulse control, reflected in SST performance, are driven by an association of faster motor response times with increasing IR.

This result is consistent with multiple studies that implicate central insulin signaling as a modulator of activity in neural circuits subserving a wide range of behaviors including cognition, impulsivity and behavioral self-regulation [3–11]). It is also consistent with the relatively small number of studies that have examined the relationship between impulsivity and markers of insulin resistance. Thus Lasselin et al. found choice reaction times in T2DM patients receiving insulin were similar to matched healthy controls, and both were longer than T2DM patients receiving no or only oral antidiabetic medications, whose HbA1c was also significantly greater, consistent with poorer insulin signaling [30]. Hawkins et al. (2016) [31] found that in otherwise healthy lean and obese adults performing a go–no-go task, higher fasting plasma glucose was a predictor of more commission errors and faster reaction times, and irrespective of BMI, those whose fasting plasma glucose was in the “pre-diabetic” range (>100 mg/dL) had significantly more commission errors. Galioto et al., found that change in HOMA-IR, but not HbA1c, predicted changes in cognitive and psychomotor scores from baseline in obese patients one year following bariatric surgery [32]. Finally, Eisenstein et al. observed that lower insulin sensitivity predicted greater delayed reward discounting in obese volunteers [33].

The weak association of SST performance with BMI in the present study is consistent with the majority of prior findings: while there is evidence for impaired inhibitory control with obesity in children and adolescents, and obese adolescents with faster inhibitory responses have better weight loss outcomes [34–37] [38], the evidence for specific obesity-associated compromise of performance in reactive motor inhibition, cognitive control or executive function in adults is mixed. While some studies report longer SSRTs in obese compared with healthy weight subjects [39–41], the majority find no difference [37, 42–47] (and see recent review by Bartholdy et al 2016 [48]). While less frequently reported, the majority of studies find no association of mean response time (MRT) or go speed with BMI [48], though Grant et al. found longer reaction times in obese compared with normal weight adults [44], and Batterink et al. found faster reaction times with increasing BMI in adolescent girls performing a food-specific Go–No-Go task [34]. In these studies, however, obese participants were described as otherwise healthy; their IR was not assessed, and its potential importance as an explanatory variable in obese individuals was not explored.

An important caveat is that the focus here is on obese volunteers with T2DM. It is possible that by including only obese volunteers we may be underpowered to detect obesity-associated contributions to behavioral and neural variability due to a limited BMI range, a feature that may also contribute to the lack of significant correlation of IR severity with BMI in this cohort. While we cannot exclude this possibility, and follow-up studies would be strengthened by the inclusion of lean volunteers, our study subjects’ BMIs were evenly distributed across a range (median BMI = 36.7, range = 21.1, interquartile range = 6.6) sufficient to have revealed significant behavioral, neural, and/or molecular associations in previous individual studies [33, 66–70] and see reviews [38, 48].

### Impact of obesity on neural correlates of SST

While a poor predictor of SST performance (cSSD; SSRT) or of activation of the strongest neural predictors of SSRT (left SFG, MFG, IFG), higher BMI was nonetheless a predictor of reduced activation in rMFG (CON_SS>GS_) in the present study. Similar associations have been found in adolescent girls performing a food-cue specific go–no-go task [34], and in women performing the SST [45]. Curiously however, these BMI-associated neural deficits were not significant predictors of motor inhibitory performance. Rather, as noted earlier, Batterink et al. found that greater BMI predicted faster response times both for go trials and no-go errors, as well as greater no-go error rates in adolescent girls [34], while Hendrick et al. found no difference in SST performance between obese and lean women, nor any decrease in activation of inhibitory networks with increasing BMI. Rather they observed a BMI-associated decrease in activation of a network of areas supporting saliency processing [45]. These findings are consistent with our own that while increased BMI was indeed associated with less rMFG activation for SS>GS, the reduced activation of right frontal cortices, including rMFG, was associated with improved rather than degraded overall SST performance (longer cSSD), a longer mGRT, and had no significant impact on SSRT. Thus it appears that with increasing BMI, activation of right frontal cortex is reduced in a manner that leads to slower “go” responses. This result was unanticipated, and indeed contrary to our initial hypothesis that greater BMI would be associated with impaired overall performance (shorter cSSD) arising from blunted activation of inhibitory areas recruited during stop trials, with a consequent increase in SSRT. Taken together with the prior behavioral and fMRI results [34, 45], our findings are consistent with a more general role for right PFC areas in rule maintenance, and contextual response selection and/or response switching rather than inhibition. Thus with increasing BMI, impaired frontal activation may slow contextual processing and response selection leading to slower go speeds (longer mGRT) and reduced overall impulsivity (longer cSSD).

### Impact of insulin resistance on neural correlates of SST

Our finding that an association of increased striatal activation with more severe IR mediated the faster go speeds observed in participants with greater HOMA-IR supports the idea that, given the broad distribution of IRs in the brain, insulin acting centrally might influence brain networks supporting a diverse repertoire of behaviors and cognitive functions. Manipulation of insulin levels, whether systemically [5, 7, 8, 71–75] or in CNS directly [3, 4, 6, 9, 76–79], modulates basal neural activity as well as the amplitude of task-associated neural activation. These effects have been observed not only in areas regulating energy homeostasis, but also more broadly in areas implicated in reward, motivation, attention and cognition (for reviews see [10] [11]).

Since the present study did not directly measure brain IR, all our behavioral and neural correlates are with HOMA-IR. This is an important caveat. Nonetheless, several lines of evidence suggest that central insulin resistance may underlie, at least in part, the observed association of increased striatal activation with increasing HOMA-IR. Obesogenic diets quickly induce insulin resistance in diverse brain areas in animal models [21, 80–82], and the severity of peripheral and central IR in these models are correlated. A causal association is suggested by the observation that peripheral insulin resistance can be modulated by changes in central insulin signaling tone in humans [4, 83]. Finally, in lean, overweight and obese volunteers, peripheral IR is a significant predictor of intranasal insulin’s ability to modulate cortical and subcortical neural activity [84], and in T2DM patients greater peripheral IR is associated with increasing disruption of brain network connectivity [85, 86]. Taken together, these findings suggest that in the present study, HOMA-IR is a reasonable surrogate marker of central IR, and while characterization of the degree of central insulin resistance was beyond the scope of this study, our finding is consistent with prior demonstrations of a direct effect of central insulin signaling on neural activity in animal models [22, 87] and humans [3, 6, 9, 76, 78, 79, 84, 88, 89].

Though only trend level (p < 0.10), the association of heightened activation of salience attribution areas with increased IR generalizes the previously reported association of IR severity with activation of the insula by salient food cues [90]. Indeed, the frequency of comorbid IR in obesity, and the now classic and robust finding of increased activation of networks supporting attention and salience attribution by obesogenic food cues in obese compared with lean participants [34, 67, 91–96], suggest that IR may contribute in a key way to obesogenic feeding behaviors by heightening the saliency of, and impulsive responding to feeding cues in the environment.

Thus it appears likely that IR rather than obesity per se may underlie, at least in part, the obesity-associated increases in impulsivity reported in previous studies: when compared with lean healthy controls, nominally “healthy” obese participants enrolled in previous studies are likely to have had varying degrees of increased IR, as well as other clinical features of metabolic syndrome.

### Central insulin resistance and impulsivity: A pathway to obesity?

Obesogenic diets induce central insulin resistance [21, 80], the severity of which is correlated with the degree of peripheral impairment, allowing HOMA-IR to serve as a surrogate [7, 12]. The impact of blunted insulin signaling on dopamine systems, the association of insulin resistance with obesity, and the compromise of dopamine signaling seen in obesity, together suggest that it may be central insulin resistance, rather than obesity per se, that compromises appropriate regulation of hedonic feeding behavior.

The present study provides support for this idea, showing that increasing IR potentiates the activation of brain areas subserving attention, salience attribution, motivation and motor speed [97–99] in a manner that leads to heightened impulsive responding. These results suggest that insulin resistance may promote goal-directed impulsive responding by potentiating activation of attention and motor networks in response to salient food cues. Consistent with this idea, the areas identified in the present study as well as those identified by Jastreboff and colleagues as IR- and/or glucose sensitive mediators of cue induced food craving in obese participants [74], correspond closely with those identified by Stoeckel and colleagues as displaying heightened activation in response to food cues compared with non-food cues in obese compared with non-obese participants [95]. Furthermore, Eisenstein and colleagues observed that in obese volunteers, blunted insulin sensitivity reduces the value of future reward, suggesting that the benefits of weight loss may be discounted to a greater extent with increasing insulin resistance [33].

This association of increased IR with increasing activation of circuits driving salience attribution and goal-directed impulsive responding, coupled with greater discounting of future rewards of weight loss suggests a vicious cycle in which consumption of obesogenic foods (which are generally more palatable and rewarding!) increases central IR, with a consequent dysregulation of DA tone leading to heightened saliency of obesogenic food cues, increased impulsive feeding, and a further increase in IR. It also provides a molecular and neural rationale for why control of food intake may be especially challenging for the obese diabetic patient: in an environment filled with salient food cues, central IR heightens the saliency of obesogenic food cues, while increased impulsivity and motivation to act on these cues is abetted by the ready availability of highly palatable obesogenic and diabetogenic foods.

### Cellular and molecular basis for impaired SST performance with insulin resistance

Insulin signaling plays an important role in setting striatal DA tone. Depressed insulin signaling in striatal DA terminals leads to reduced DAT surface expression, blunting DA clearance [16, 87], altering reward learning behavior and distorting motivational salience [19, 100, 101]. Decreased availability of striatal D2 dopamine receptors with increasing BMI is a feature of morbid (BMI > 40) obesity [102], and is a predictor of decreased activity in frontal cortical areas subserving cognitive processes related to impulse control, response selection, error detection and performance monitoring [103].

Psychomotor speed, an important component of impulsivity [27], is heavily dependent on striatal DA [23–26]: dorsal striatal D2R antagonism slows go responses [28] while elevated synaptic DA facilitates them [104], and promotes heightened impulsivity in humans [29]. This suggests that obesogenic/diabetogenic diets may, by increasing central IR, blunting striatal DA clearance and increasing DA tone, heighten impulsivity by increasing psychomotor speeds. Our results support such a model: IR but not BMI, was the principal predictor of SST performance in obese volunteers with T2DM, and its impact was to increase go speed, consistent with an IR-associated hyper-DAergic state, an association mediated by the impact of IR on activation of a striato-cortico-thalamic network that was itself a predictor of go speed.

## Conclusions

Notwithstanding the compelling body of literature confirming the importance of fronto-striatal networks as the neural correlates of inhibitory control, our results suggest that in the setting of obesity-associated T2DM, it is the impact of insulin resistance on brain networks driving go speeds (mGRT), rather than that of BMI on networks driving stop speeds (SSRT), that distinguishes obese individuals with poorer (shorter cSSD) from those with better overall impulse control (longer cSSD). This increased motor impulsivity is mediated by an IR-associated increase in excitability of dorsal striatum within broader cortico-striatal-thalamo-cortical networks subserving attention, salience attribution, action selection and cognitive control. These findings are consistent with the idea that insulin signaling may act in the central nervous system to tune dopamine tone in circuits central to the reward learning, attribution of salience, and response selection, the distortion of which by central insulin resistance, may increase the salience, impulsive seeking and consumption of the very obesogenic/diabetogenic foods that drive insulin resistance. Finally, the present study, suggests that the impact of impairments in insulin signaling in obese populations cannot be discounted, and future studies of the influence of obesity on brain-behavior relationships should control for IR.

## Supporting information

S1 File **Table A: Stop Signal Task (SST). Parent (Behavioral) Group:** volunteers whose SST performance met criteria for inclusion in behavioral analyses (see Methods). **fMRI subgroup:**volunteers meeting SST performance criteria whose fMRI head motion met criteria for inclusion in fMRI analyses. All data mean ± SD.
**Table B: Impulsivity, Inhibitory, and Error Monitoring Circuits in the Stop Signal Task: Neural correlates and sensitivity to BMI and/or HOMA-IR.** Areas identified as potential neural predictors of SST performance based on whole brain voxel-wise regression of activation contrast (SS>GS) with mGRT and SSRT. Brain regions and associated t-statistic, cluster sizes, and MNI coordinates are from the location of peak voxel at each local cluster maxima. Regions thresholded at p_unc_<0.005, k_E_>10 voxels. ^†^ HOMA-IR is a predictor of contrast strength. * BMI is a predictor of contrast strength.
**Figure A: The Stop Signal Task. A)** The stop signal task in an fMRI design where the green circle begins each trial and is preceded by a variable-length fore-period. In stop trials, the red **X** is presented following a variable stop signal delay. A button press on a go trial is a go success (GS) while failing to press the button on a go trial is a go error (GE). Inhibiting the button press on a stop trial is a successful stop (SS) while pressing the button on a stop trial is a stop error (SE). **B)** The horse race model assumes that go and stop processes are independent where the inhibitory response (stop signal response time) is calculated by subtracting the critical stop signal delay (time between “go” signal [green circle] and “stop” signal [red X]) from the median go response time. SSRT—stop signal response time; SSD—stop signal delay; mGRT—median go response time.
**Figure B: Neural predictors of SST performance.** Voxel-wise whole brain regression identified areas having significant association of SS-GS contrast (CON_SS>GS_) with **A)** mGRT; **B)** SSRT. Faster go responses (shorter mGRT) were associated with greater activation of a largely right hemisphere network of motor control and attentional regions (see Table B) including precentral gyrus, supplementary motor area, middle frontal gyrus, precuneus, angular gyrus and thalamus, but less activation in left IFG/ventral insula, middle temporal gyrus, and amygdala. Faster stopping was associated with increased activation in the left inferior, middle, and superior frontal gyri and less activation in the supramarginal gyrus and the insula. Figures thresholded at p_unc_<0.001, k_E_>10 voxels.
**Figure C: Association of strength of activation of a cortico-striatal network with insulin resistance mediates the faster go speeds (shorter mGRT) observed with greater insulin resistance in obese T2DM participants.** Cortico-striatal circuits subserving impulsivity (red ROIs whose activation contrast predicts mGRT) mediate HOMA-IR’s effect on mGRT in the SST. HOMA-IR was at least a trend level predictor of activation (a) in highlighted striatal and frontal cortical areas for which whole brain voxel-wise regression confirmed activation strength was a predictor of mGRT SST (b), and these paths (axb) were significant mediators of the association of faster go speeds (shorter mGRT) with increasing insulin resistance (HOMA-IR). Table shows associated path coefficients, with 95% confidence intervals determined by bootstrapping, and p value.
(PDF)
