# Supplementary material for: An insulin resistance associated neural correlate of impulsivity in type 2 diabetes mellitus
Source: PLoS One. 2017 Dec 11;12(12):e0189113. doi: 10.1371/journal.pone.0189113 (PMC5724830; doi:10.1371/journal.pone.0189113)
Supplement: S1 File — Table A: Stop Signal Task (SST). Parent (Behavioral) Group: volunteers whose SST performance met criteria for inclusion in behavioral analyses (see Methods). fMRI subgroup:volunteers meeting SST performance criteria whose fMRI head motion met criteria for inclusion in fMRI analyses. All data mean ± SD. Table B: Impulsivity, Inhibitory, and Error Monitoring Circuits in the Stop Signal Task: Neural correlates and sensitivity to BMI and/or HOMA-IR. Areas identified as potential neural predictors of SST performance based on whole brain voxel-wise regression of activation contrast (SS>GS) with mGRT and SSRT. Brain regions and associated t-statistic, cluster sizes, and MNI coordinates are from the location of peak voxel at each local cluster maxima. Regions thresholded at punc<0.005, kE>10 voxels. † HOMA-IR is a predictor of contrast strength. * BMI is a predictor of contrast strength. Figure A: The Stop Signal Task. A) The stop signal task in an fMRI design where the green circle begins each trial and is preceded by a variable-length fore-period. In stop trials, the red X is presented following a variable stop signal delay. A button press on a go trial is a go success (GS) while failing to press the button on a go trial is a go error (GE). Inhibiting the button press on a stop trial is a successful stop (SS) while pressing the button on a stop trial is a stop error (SE). B) The horse race model assumes that go and stop processes are independent where the inhibitory response (stop signal response time) is calculated by subtracting the critical stop signal delay (time between “go” signal [green circle] and “stop” signal [red X]) from the median go response time. SSRT—stop signal response time; SSD—stop signal delay; mGRT—median go response time. Figure B: Neural predictors of SST performance. Voxel-wise whole brain regression identified areas having significant association of SS-GS contrast (CONSS>GS) with A) mGRT; B) SSRT. Faster go responses (shorter mGRT) were associate [file pone.0189113.s001.pdf]

Figure A

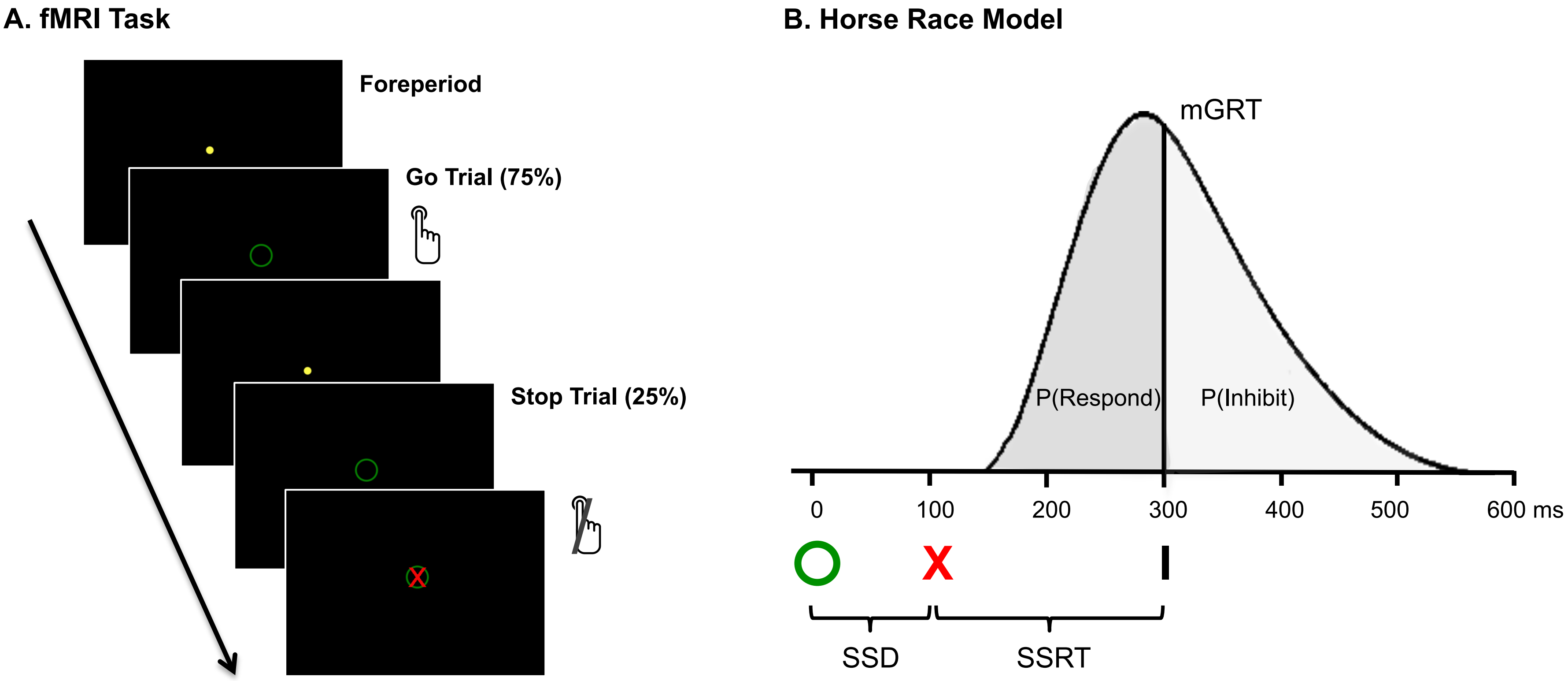
A. fMRI Task
Foreperiod
Go Trial (75%)
Stop Trial (25%)
B. Horse Race Model
mGRT
P(Respond)
P(Inhibit)
0
100
200
300
400
500
600 ms
SSD
SSRT

Table A

	Parent (Behavioral) Group	fMRI Sub-group	p_{mean} [a]	p_{var} [a]
N	47	30	-	
cSSD (ms)	310 ± 119<br>[236 315 408]	319 ± 127<br>[241 323 437]	0.48	0.40
mGRT (ms)	605 ± 107<br>[520 595 675]	620 ± 115<br>[548 595 730]	0.21	0.22
SSRT (ms)	295 ± 32<br>[276 295 319]	301 ± 30<br>[279 301 320]	0.12	0.40

Figure B

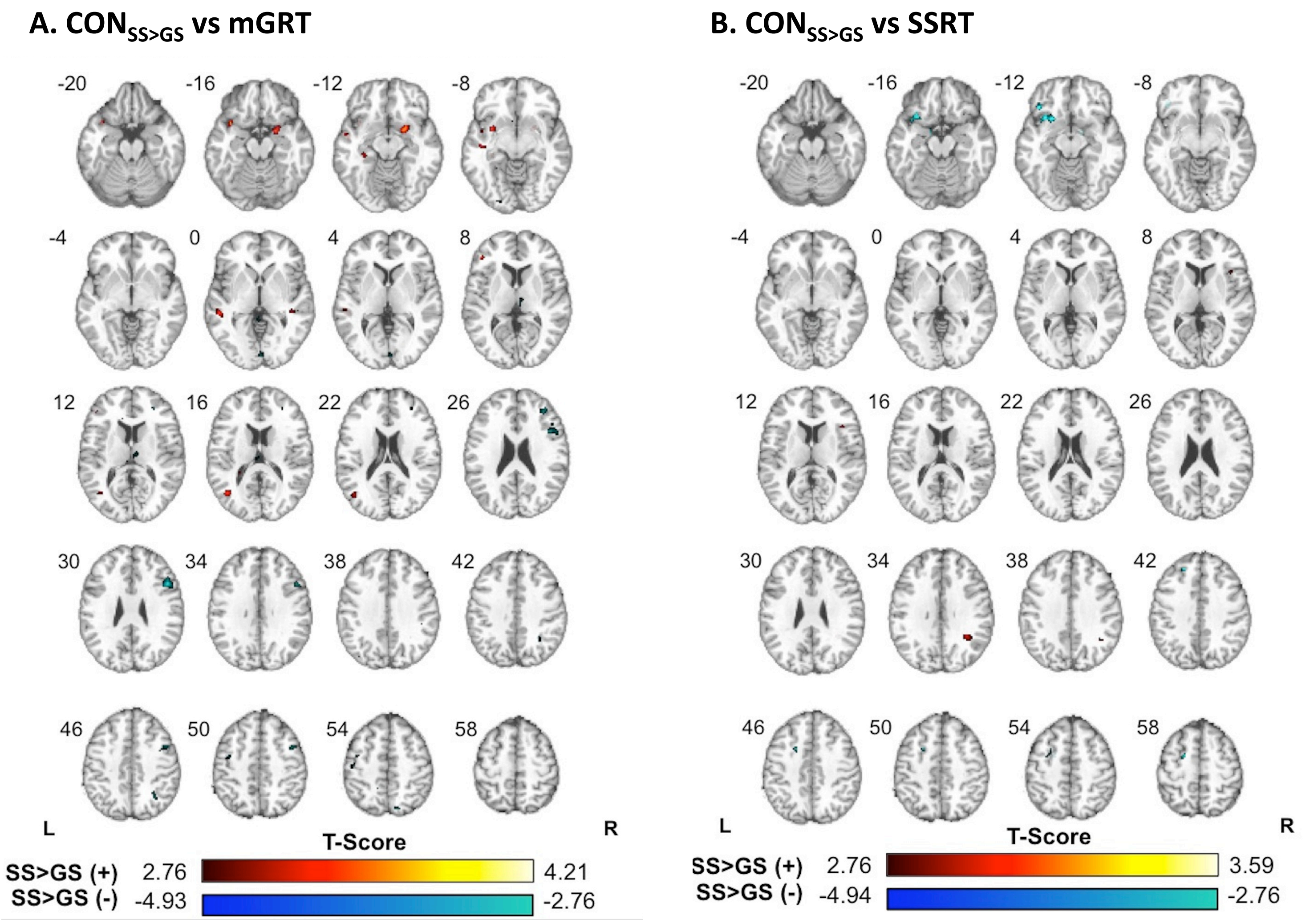
A. CON$_{SS>GS}$ vs mGRT
-20
-16
-12
-8
-4
0
4
8
12
16
22
26
30
34
38
42
46
50
54
58
L
R
T-Score
SS>GS (+) 2.76 4.21
SS>GS (-) -4.93 -2.76
B. CON$_{SS>GS}$ vs SSRT
-20
-16
-12
-8
-4
0
4
8
12
16
22
26
30
34
38
42
46
50
54
58
L
R
T-Score
SS>GS (+) 2.76 3.59
SS>GS (-) -4.94 -2.76

Table B

Neural Correlates of SST Performance: $CON_{SS>GS}$ vs median Go Response Time (mGRT), Stop Signal Response Time (SSRT)

Regressor	Brain Region	Hemi.	Voxels	T-Statistic	x	y	z	R	p
mGRT	Angular Gyrus	R	16	3.3998	33	-58	43	-0.548	0.002
	Cuneus	L/R	13	3.6767	3	-88	1	-0.530	0.003
	Middle Frontal Gyrus*	R	21	3.7815	36	38	25	-0.608	<0.001
		R	57	4.216	48	17	28	-0.620	<0.001
	Precentral Gyrus†	R	21	3.5566	42	5	49	-0.552	0.002
	Precuneus†	R	11	3.362	15	-73	52	-0.513	0.003
	Supplementary Motor Area†	L	25	3.6703	-6	-10	67	-0.560	0.001
	Thalamus†	L/R	20	3.1663	9	-16	10	-0.528	0.001
	Amygdala	R	28	4.9367	21	2	-14	0.652	<0.001
	Inferior Frontal Gyrus	L	12	4.4576	-36	11	-17	0.624	<0.001
		L	11	3.984	-45	38	10	0.482	0.007
	Insula	L	10	3.7112	-45	-16	-8	0.576	0.001
	Middle Temporal Gyrus	L	30	3.8062	-39	-64	16	0.582	0.001
		L	25	4.109	-51	-31	1	0.611	<0.001
		L	16	3.8012	-54	-1	-8	0.580	0.001
	Putamen	L	11	3.8772	-30	5	-8	0.578	0.001
SSRT	Supramarginal Gyrus	R	24	3.5862	33	-49	31	0.592	0.001
	Insula	R	12	3.4889	39	17	7	0.703	<0.001
	Superior Frontal Gyrus	L	11	4.0994	-18	-1	55	-0.711	<0.001
	Middle Frontal Gyrus	L	13	3.5829	-21	8	46	-0.684	<0.001
	Inferior Frontal Gyrus	L	46	4.5674	-30	17	-14	-0.551	0.002
		L	17	4.9397	-39	29	-14	-0.611	<0.001

Figure C

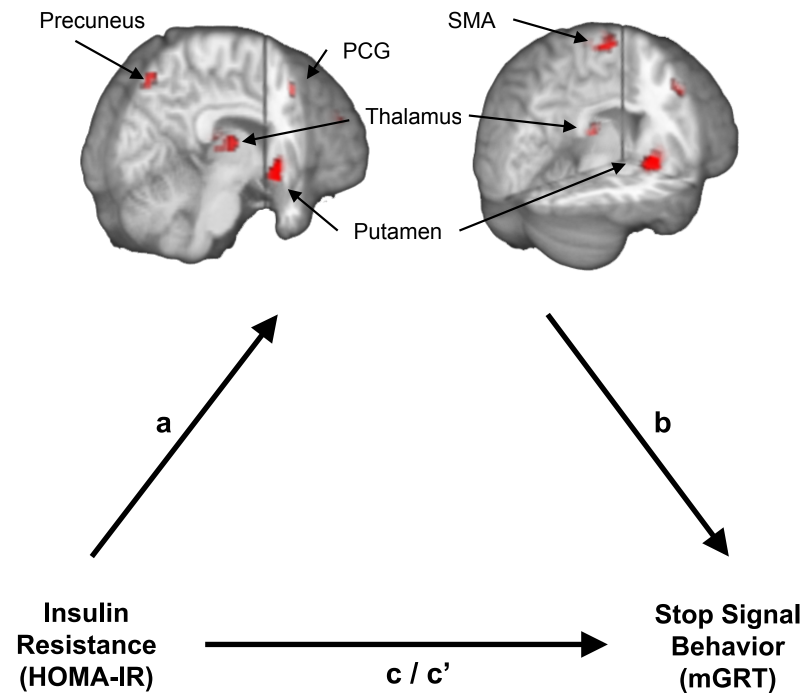

**Path**<br>effect [95% CI]<br>(p-value)	**a**<br>HOMA-IR → activation	**b**<br>activation → mGRT	**c (total)**<br>HOMA-IR → mGRT	**c’ (direct)**<br>HOMA-IR → mGRT controlling for activation	**a x b (indirect)**<br>HOMA-IR → mGRT mediated by activation<br>*K2 [95% CI] – Effect Size (S, M, L)*
Putamen (R)	0.27 [-0.044, 0.59]<br>(0.09)	-17.2 [-23.95, -10.45]<br>(<0.0001)	-9.65 [-17.57, -1.74]<br>(0.019)	-4.96 [-10.81, 0.88]<br>(0.09)	-4.69 [-11.53, -0.91]<br>(0.09)<br>0.20 [0.037, 0.38] (M)
Precuneus (R)	0.67 [0.23, 1.12]<br>(0.004)	-4.18 [-10.28, 1.92]<br>(0.17)	-9.65 [-17.57, -1.74]<br>(0.019)	-6.84 [16.09, 2.40]<br>(0.14)	-2.81 [-9.63, 2.92]<br>(0.21)<br>0.10 [0.007, 0.30] (S-M)
SMA (L)	0.19 [0.060, 0.33]<br>(0.007)	-18.17 [-40.26, 3.93]<br>(0.10)	-9.65 [-17.57, -1.74]<br>(0.019)	-6.13 [-14.00, 1.74]<br>(0.12)	-3.52 [-10.13, -0.41]<br>(0.159)<br>0.13 [0.016, 0.32] (M)
Thalamus (R)	0.37 [-0.010, 0.76]<br>(0.056)	-6.61 [-14.86, 1.638]<br>(0.11)	-9.65 [-17.57, -1.74]<br>(0.019)	-7.185 [-14.85, 0.484]<br>(0.07)	-2.47 [-8.71, 0.28]<br>(0.24)<br>0.10 [0.007, 0.304] (S-M)
Precentral Gyrus (R)	0.31 [0.082, 0.54]<br>(0.01)	-10.8 [-25.01, 3.45]<br>(0.14)	-9.65 [-17.57, -1.74]<br>(0.019)	-6.28 [-14.70, 2.13]<br>(0.14)	-3.37 [-10.52, 1.52]<br>(0.19)<br>0.13 [0.014, 0.33] (M)
